# Symbiotic bacterial communities in rainforest fungus-farming ants: evidence for species and colony specificity

**DOI:** 10.1038/s41598-020-66772-6

**Published:** 2020-06-23

**Authors:** Mariane U. V. Ronque, Mariana L. Lyra, Gustavo H. Migliorini, Maurício Bacci, Paulo S. Oliveira

**Affiliations:** 1 Programa de Pós-Graduação em Ecologia, Instituto de Biologia, C.P. 6109, Universidade Estadual de Campinas, 13083-862 Campinas, SP Brazil; 2 Departamento de Biodiversidade, Instituto de Biociências, Universidade Estadual Paulista - Campus Rio Claro, 13506-900 Rio Claro, SP Brazil; 3 Programa de Pós-Graduação em Biologia Animal, Universidade Estadual Paulista - Campus São José do Rio Preto, 15054-000 São José do Rio Preto, SP Brazil; 4 Centro de Estudos de Insetos Sociais, Universidade Estadual Paulista - Campus Rio Claro, 13506-900 Rio Claro, SP Brazil; 5 Departamento de Biologia Animal, C.P. 6109, Universidade Estadual de Campinas, 13083-862 Campinas, SP Brazil

**Keywords:** Microbial ecology, Entomology

## Abstract

Animals may host diverse bacterial communities that can markedly affect their behavioral physiology, ecology, and vulnerability to disease. Fungus-farming ants represent a classical example of mutualism that depends on symbiotic microorganisms. Unraveling the bacterial communities associated with fungus-farming ants is essential to understand the role of these microorganisms in the ant-fungus symbiosis. The bacterial community structure of five species of fungus-farmers (non-leaf-cutters; genera *Mycocepurus*, *Mycetarotes*, *Mycetophylax*, and *Sericomyrmex*) from three different environments in the Brazilian Atlantic rainforest (lowland forest, *restinga* forest, and sand dunes) was characterized with amplicon-based Illumina sequencing of 16 S ribosomal RNA gene. Possible differences in bacterial communities between ants internal to the nest (on the fungus garden) and external foragers were also investigated. Our results on the richness and diversity of associated bacteria provide novel evidence that these communities are host- and colony-specific in fungus-farming ants. Indeed, the bacterial communities associated with external foragers differ among the five species, and among colonies of the same species. Furthermore, bacterial communities from internal ants vs. foragers do not differ or differ only slightly within each ant species. This study highlights the importance of describing ant-associated bacterial communities to better understand this host-bacterial interaction in the social environment of insect colonies and provides the foundation for future studies on the ecological and evolutionary processes that drive the success of fungus-farming ants.

## Introduction

Symbiotic microorganisms play crucial roles in shaping the phenotypes, ecology, and evolution of their hosts^1,2^. Advances in DNA sequencing techniques and culture-independent genomic tools have resulted in accurate estimates of microbial richness and diversity from host and environmental samples, revealing an abundant and diverse microbiota living in symbiosis with animals^1,3^. These microorganisms may interact with hosts in many ways, such as preventing pathogenic infections, increasing the host’s ability to cope with stressful environments, or even degrading or synthesizing substances that are important for host nutrition^2,4,5^. According with the “holobiont” concept, the biological entity on which selection acts is the host and its associated microorganisms together as a unit^2^.

Fungus-farming ants (Formicidae, Myrmicinae, Attina; hereafter referred to as attine ants) are a good example of the tight links between symbiotic microorganisms and host evolutionary success. These ants live in a complex and highly specialized multi-trophic symbiosis^6^. As an example of obligate mutualism, ants obtain food for the colony by cultivating certain fungal species in their nests, and in return the ants provide the fungus with nourishment, dispersal to new locations, and an environment free of parasites and competitors, such as parasitic fungi from the genus *Escovopsis*^7^. Attine ants are divided into two clades, the Paleoattina and the Neoattina^8^. The latter includes the highly derived attine genera *Acromyrmex* and *Atta*, known as “leaf-cutter ants” due their behavior of cutting fresh leaves as the substrate on which they cultivate the fungus garden^6^. *Acromyrmex* and *Atta* have the most derived characteristics within fungus-growers and many studies investigate the bacterial communities associated with these ants^9–11^.

The bacterial community in other attine genera from Paleoattina and Neottina (non-leaf-cutter ants) is poorly known^12^. Some bacteria present on the ant body surfaces and within the fungus garden are believed to aid in managing the fungiculture and avoiding infection by *Escovopsis*^13–16^, although little is known about their interactions within the nest environment. For instance, these bacteria could be nutritionally important^12^, play a defensive role against pathogens by producing antibiotic compounds^16,17^, or even contribute to nest hygiene^18^. In addition, the fungus garden is constantly exposed to external bacteria that are brought to the nest by ant foragers and/or with items they collect as substrate for fungiculture^18^, but the influence of this continuous exchange on the nest environment is unknown. Comprehensive studies of these ant- and nest-associated bacterial communities are important to reveal the bacterial community composition of fungus-farming ants and their nests, as a first step in understanding the role of these bacteria in ant-fungus symbiosis.

In this study, we report on the diversity of the bacterial communities associated with five species of fungus-farming ants (non-leaf-cutters) from three different areas in the Atlantic rainforest of Brazil: *Mycocepurus smithii* (Forel, 1893), *Mycetarotes parallelus* (Emery, 1906), *Mycetophylax morschi* (Emery, 1888), *Sericomyrmex parvulus* (Forel, 1912) and *Sericomyrmex saussurei* (Emery, 1894). We quantified bacterial species richness and diversity using 16 S ribosomal RNA gene amplicon sequencing. Our objectives were: (i) to characterize the bacterial community composition from five attine species; (ii) to compare the microbiome of the five attine species and investigate possible interspecific differences in bacterial community composition; (iii) to investigate if the microbiota associated with ants whose activity is internal to the nest environment differs from that associated with ants foraging outside the nest; and (iv) to compare the microbiome between different colonies of the same species and investigate possible intraspecific differences in the composition of associated bacterial communities. Although the five ant species investigated cultivate mutualistic fungi, they differ in the size of their colonies, in the material collected for fungiculture, and in the foraging modes^19^. Thus, we predicted that bacterial communities would differ among the five species of fungus-farming ants. Because the environment outside the nest has several sources for transient bacteria (e.g., soil, plants, other arthropods, material collected for fungiculture), we expected that ant foragers would have different bacterial composition when compared to ants working inside the nest. Our results provide novel evidence that ant-associated bacteria are host- and colony-specific in fungus-farming ants. Bacterial communities associated with external foragers differ among the five species, and among colonies of the same species, suggesting that environment outside the nest has little influence on the associated bacterial community of fungus-farming ants.

## Material and methods

### Samplings

We carried out fieldwork in July 2015 and January 2016 in the Atlantic rainforest at the ‘Parque Estadual Serra do Mar’, Ubatuba municipality, São Paulo State, Brazil (Fig. S1 Supplementary Material 1). We collected workers of five species of fungus-farming ants: *Mycocepurus smithii*, *Mycetarotes parallelus*, *Mycetophylax morschi*, *Sericomyrmex parvulus* and *Sericomyrmex saussurei*. Sampled colonies were located in three different areas: (i) *S. parvulus* and *S. saussurei* were found in lowland forest (23° 21’ 52.1” S, 44° 49’ 28.5” W), characterized by clay-sandy soils, epiphytes, and trees reaching more than 20 m^20^; (ii) *Mycocepurus smithii*, *Mycetarotes parallelus* and *Mycetophylax morschi* were found in *restinga* forest (23° 21’ 28.4” S, 44° 51’ 00.7” W) growing in sandy soils and with trees up to 20 m tall^20^; (iii) *Mycetophylax morschi* was also found in the dunes region (23° 21’ 38.8” S, 44° 50’ 58.1” W), this area has a xerophytic vegetation and are under the influence of sea salinity^20^. Information about the weather of the area is provided in Fig. S1 legend (Supplementary Material 1).

To investigate if bacterial communities differed between foragers in the external environment and ants working inside the nest, for *Mycocepurus smithii*, *Mycetarotes parallelus* and *Mycetophylax morschi*, we collected 16 foraging workers upon their return to the nest and 16 workers from the fungus garden inside the nest. Due to difficulties in finding the nest chambers of *Sericomyrmex*, only foragers were collected for *S. parvulus* and *S. saussurei*. Workers internal to the nest were collected directly from gardens in the field, upon excavation of the colonies. Previous work^21^ with captive colonies confirmed intracolonial division of labor in these five species. Hence, at any given time, workers performing foraging activities do not sequentially engage in work on the fungus garden, and vice-versa. Thus, in this study we consider two non-mixing worker groups: those collected *on the fungus garden* and those collected as *outside foragers* (collected upon their return to the nest).

The following colonies of ants were sampled: three colonies of *Mycocepurus smithii*, *Mycetarotes parallelus*, and *Mycetophylax morschi* (*restinga* forest); three colonies of *S. parvulus* (lowland forest); two colonies of *Mycetophylax morschi* (dune area); and four colonies *S. saussurei* (lowland forest). All equipment used for collecting ants was sterilized with ethanol 70% and flamed to avoid sample contamination. Ants were stored in separate sterile microcentrifuge vials, in a −20 °C freezer. Since generic names begin with the same letters in the species *Mycocepurus smithii*, *Mycetarotes parallelus* and *Mycetophylax morschi*, the names of these species will be cited in full.

### Molecular methods

We extracted Genomic DNA from whole individual ants using the Qiagen DNeasy Blood & Tissue kit, following the manufacturer’s protocol with minor adjustments to increase DNA yield from the bacterial community. Due to the small body length of workers (<5 mm), we extracted DNA from the whole individual^4,9,12,16^. Ants were not macerated before being placed in AL buffer and incubated at 56 °C with proteinase K for 2 hours to minimize extraction of ant DNA. For the bacterial community profiling, we PCR-amplified the V4 region of the bacterial 16 S rRNA gene using a dual-index approach^22^ with barcoded-primers 515 F and 806 R^23^. PCR reactions were performed in duplicates using conditions described in Bletz *et al*.^24^, but with Phire® Hot Start II DNA Polymerase (Finnzyme, Espoo, Finland). Negative controls for extraction and each PCR mix were included to check for contamination. PCR products of each sample were combined; samples were pooled and purified using a DNA Gel Extraction Kit (Norgen Biotek Corp, Thorold, ON, Canada). The purified pool was sequenced with paired-end 250 on an Illumina MiSeq sequencer at TUCF Genomics, Boston, MA, USA, using the V4-specific primer sequences and linkers^22^.

To confirm the identity of the sampled ants of each colony, we sequenced a fragment of the mitochondrial cytochrome c oxidase subunit 1 (*COI*) using primers LCO1490 and HC02198^25^ and standard PCR conditions. PCR fragments were purified and sequenced in Macrogen Inc, Seoul, South Korea. Sequences were trimmed and quality-checked using Geneious R6 (http://www.geneious.com ^26^) and submitted to GenBank (accession numbers MH206536- MH206586). We obtained good sequences from ants from all colonies and found almost no variation between colonies and within species. *Mycocepurus smithii*, *Mycetarotes parallelus* and *S. parvulus* presented only one haplotype in all colonies, and *Mycetophylax morschi* and *S. saussurei* presented two haplotypes that differ in only two base pairs. We found no variation between individuals from the same colony.

### Sequence analyses

The sequences were first demultiplexed and converted in fastq format using Illumina’s bcl2fastq conversion software (Illumina, San Diego, CA, USA). Then we processed the reads and analyzed the data using QIIME 2 2020.2^27^ on Ubuntu 18.04.4 LTS. We imported the demultiplexed read pairs into QIIME 2, merged pair-ends with VSEARCH^28^, quality filtered using q-score-joined, and used Deblur^29^ to denoise data with a trimming length of 250 nt. OTUs comprising less than 10 reads in total were filtered out. Sequences were aligned with mafft^30^ and the FastTree2^31^ was used to build a phylogenetic tree of OTUs using QIIME 2 standard procedures. We assigned taxonomy to OTUs using the q2-feature-classifier classify-sklearn^32^ against the Greengenes 13.8, with 99% similarity with the OTUs reference sequences (from 515 F/806 R region of the sequences^33^). We rarefied all samples to 2000 reads. After filtering, 180 samples remained for analysis out of 243. See Table S1 (Supplementary Material 1) for an overview of sample sizes and numbers of sequences for different subsets of data used in the analysis. One extraction control was retained after quality filtering and rarefaction, however, this control was different from all samples of the dataset and was filtered out from diversity analyses. We also numbered the OTUs that were different, but that had the same classification until taxonomic level of genus (e.g., *Pseudonocardia* OTU 1 and *Pseudonocardia* OTU 2 are different OTUs, but from the same genus).

### Statistical analyses

We first performed descriptive analyses of bacterial community structure of the five species of fungus-farming ants. We represented by Venn diagrams (generated by Bioinformatics & Evolutionary Genomics^34^) the OTUs that were shared between all species, and between ants internal and external to the nest. We also constructed a heatmap to indicate the variation of relative abundance of bacterial OTUs in all species, discriminating the external and internal ants. The heatmap was generated in R version 3.3.3^35^ with the package *gplots* (function *heatmap.2*), and the dendrograms were generated with Bray-Curtis distance matrices (*vegan* package, function *vegdist*). We removed the OTUs with less than 5% relative abundance.

All statistical analysis of alpha and beta diversity was performed in R version 3.3.3^35^. We used QIIME 2 to calculate the number of observed OTUs, Chao1 and Faith’s Phylogenetic Diversity for all samples as a proxy for bacterial richness and diversity. We used linear mixed-effects models (*nlme* package, function *lme*) to investigate the variation in Faith’s Phylogenetic Diversity in OTUs from all species from which we sampled foragers external to the nest; we included species as the main explanatory variable and location as a random factor. For species from which we sampled ants internal and external to the nest (*Mycocepurus smithii*, *Mycetarotes parallelus* and *Mycetophylax morschi*), we included species and internal/external to the nest as main explanatory variables, and location as a random factor. Since *Mycetophylax morschi* was found in both the *restinga* forest and in the dunes area, ants from each site were treated separately. We performed pairwise comparisons between all species from which we sampled worker foragers external to the nest (*stats* package, function *pairwise.t.test*), and between internal and external ants of each species (*lsmeans* package, function *lsmeans)*.

For beta-diversity, we used PERMANOVAs with package *vegan* (function *adonis*) to quantify the composition similarities between bacterial communities within groups of individuals using abundance data with Bray-Curtis distances. We also conducted this analysis using the unweighted and weighted UniFrac metrics^36^ for interspecific comparisons and between ants working inside the nest and foragers. For interspecific comparisons, we considered all species from which we sampled worker foragers external to the nest, with species as the main explanatory variable. We separated *Mycetophylax morschi* data for the *restinga* forest and the dune area. For comparisons between ants working inside the nest and foragers, we considered only species with internal and external ant samples (*Mycocepurus smithii*, *Mycetarotes parallelus* and *Mycetophylax morschi* from both the *restinga* forest and the dune area), with species and internal/external to the nest as main explanatory variables. We performed pairwise comparisons with the Bray-Curtis similarity method and Bonferroni adjustment (package *EcolUtils*, function *adonis.pair*) between all species for which we sampled worker foragers external to the nest, and between internal and external ants of each species. For intraspecific comparisons, we performed PERMANOVAs for each species, considering the colony as the main explanatory variable. In this analysis, only *Mycetophylax morschi* from the *restinga* forest was considered. We used principal coordinate analysis (PCoA) to visualize the bacterial community composition using abundance data with Bray-Curtis distances, with *ape* package (function *pcoa*).

Additionally, we identified the OTUs responsible for the patterns in bacterial community internal and external to the nest of each species using similarity percentage analysis (SIMPER), based on Bray-Curtis distances with *vegan* package (function *simper*). SIMPER showed the contribution of each OTU to the dissimilarity between the internal and external communities of each species. This analysis was calculated only for species that showed differences in composition of bacterial communities between ants internal and external to the nest environment.

### Ethics approval

Samples were collected under permit by SISBIO #45317-3 and this research was registered in the SISGen platform (SISGEN #A210679). All applicable institutional and/or national guidelines for care and use of animals were followed.

## Results

### General patterns of OTU diversity

**Figure 1:**
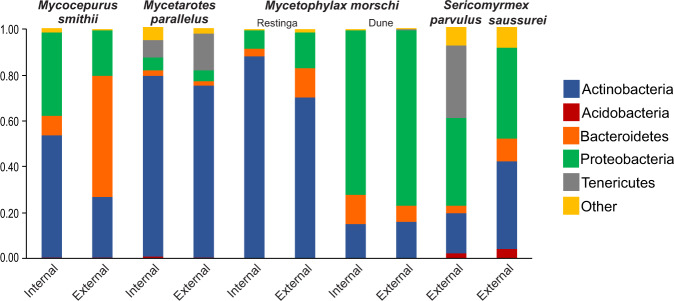
Frequency of most abundant bacterial phyla found in the five species of fungus-farming ants from the Atlantic rainforest of Brazil, for ants inside (on the fungus garden) and outside (foragers) the nest. All samples were rarefied at 2000 sequences per sample.

The analyzed data included 7,195,350 bacterial sequences from 180 samples of five species of attine ants: *Mycetarotes parallelus*, *Mycetophylax morschi*, *Mycocepurus smithii*, *Sericomyrmex parvulus* and *Sericomyrmex saussurei*. Sequences represented 874 OTUs dominated by members of the phyla Actinobacteria, Proteobacteria, Bacteroidetes, and Tenericutes (Fig. 1).

The bacterial taxonomic composition of *Mycetarotes parallelus* consisted predominantly of Actinobacteria (78.7% ants internal to nest; 75.2% external foragers). External foragers of *Mycocepurus smithii* were hosted mainly of Bacteroidetes (52.8%), whereas individuals internal to the nest had mainly Proteobacteria (36.4%) and Actinobacteria (53.7%) (Fig. 1). *Mycetophylax morschi* from *restinga* forest had predominantly Actinobacteria (88% internal; 70.2% external) and from the dune area had Proteobacteria (72.1% internal; 76.7% external). Tenericutes (31.8%) and Proteobacteria (38.4%) were the most abundant phyla in *S. parvulus* external foragers, while external ants of *S. saussurei* hosted mainly Proteobacteria (39.5%) and Actinobacteria (38.3%) (Fig. 1). The phyla Actinobacteria and Proteobacteria were present in the five ant species, both in ants inside the nest and external foragers.

Venn diagrams between the five fungus-farming ant species from which we sampled external foragers showed that 88 OTUs were common among these species (Fig. S2 Supplementary Material 1). Comparisons of the bacterial composition between ants in the internal and external nest environment, for each ant species, showed that in *Mycocepurus smithii* internal and external ants shared 35.7% of the total bacterial OTUs, *Mycetarotes parallelus* shared 44% OTUs, and *Mycetophylax morschi* from the *restinga* forest and dune area, 29.4% and 32.1% OTUs, respectively (Fig. S3 Supplementary Material 1).

**Figure 2:**
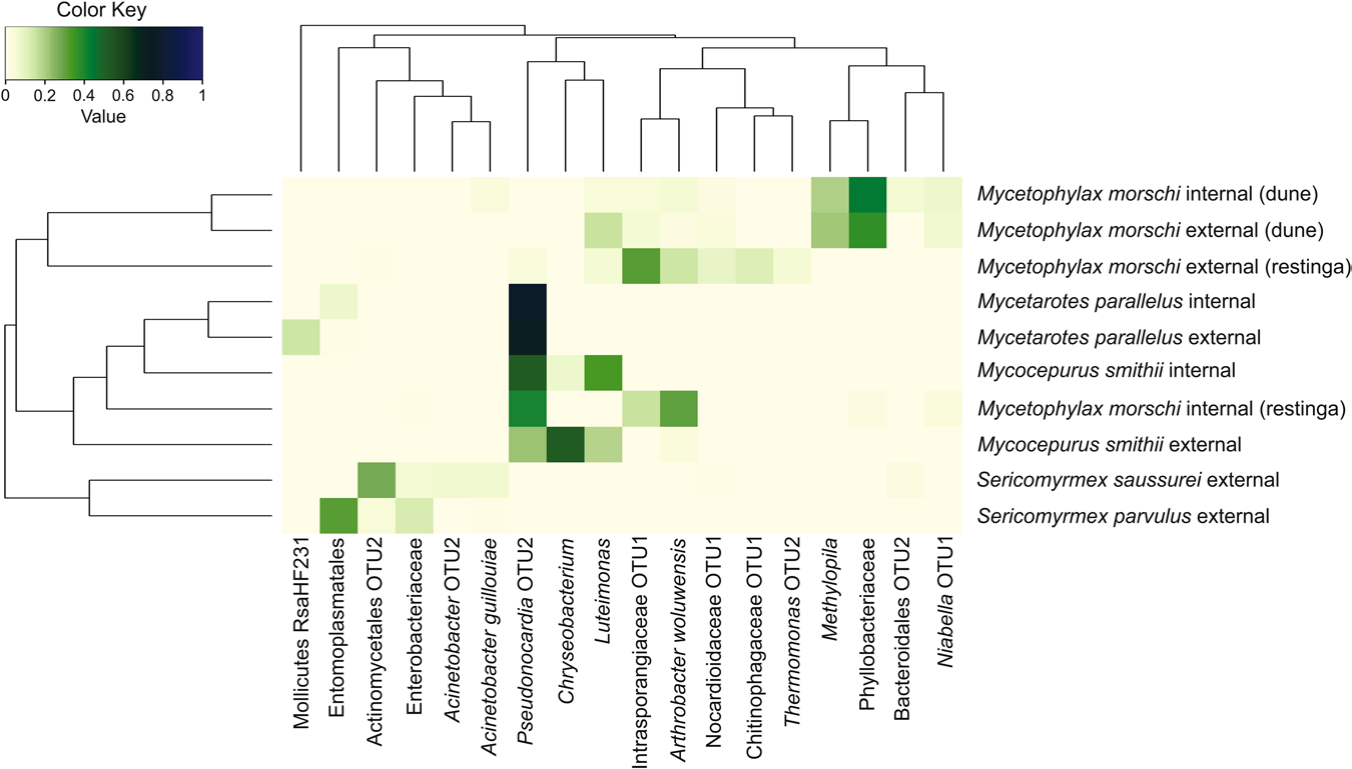
Heatmap indicating variation in relative abundance of different bacteria in the five species of fungus-farming ants from which external foragers and internal ants (on the fungus garden) were sampled in the Atlantic rainforest of Brazil. Colors indicate the relative abundance of bacterial OTUs, ranging from 0% (light yellow) to 100% (dark blue). Dendrograms were generated with Bray-Curtis distance matrices. For clarity, we removed the OTUs with less than 5% relative abundance.

The average relative abundance of bacterial OTUs in external foragers showed that different ant species were dominated by different genera of bacteria (Fig. 2). For instance, in *Mycetarotes parallelus* more than 50% of the bacterial community consisted of *Pseudonocardia* OTU 2 (Fig. 2). *Pseudonocardia* OTU 2, *Chryseobacterium* and *Luteimonas* were the main OTUs found in *Mycocepurus smithii* (Fig. 2). Workers of *Mycetophylax morschi* in the internal and external nest environment from the *restinga* forest and dune area showed that OTUs with higher average relative abundance were only found in this species. Finally, *Sericomyrmex parvulus* was dominated by Entomoplasmatales and *S. saussurei* by Actinomycetales OTU 2.

### Bacterial alpha-diversity

Within the five species for which we sampled worker foragers external to the nest, the diversity of bacterial communities of ants was higher in *S. saussurei* (followed by *S. parvulus*) as seen by richness (number of OTUs), Chao 1 diversity index and Faith’s Phylogenetic Diversity index (Table 1). For workers sampled inside the nest, *Mycetarotes parallelus* had the most diverse bacterial community, with more OTUs per sample, and higher values of Chao 1 and Faith’s Phylogenetic Diversity (Table 1).

**Table 1:** Number of bacterial OTUs, Chao1 diversity index, and Faith’s Phylogenetic Diversity index for five species of fungus-farming ants studied in Atlantic rainforest, Brazil (mean ± standard deviation; N = number of samples).

	External			Internal		
	OTUs	Chao 1	Faith’s Phylogenetic Diversity	OTUs	Chao 1	Faith’s Phylogenetic Diversity
*Mycocepurus smithii*	34.47 ± 17.76 (N = 21)	65.22 ± 43.62	5.94 ± 2.68	42.10 ± 20.74 (N = 20)	56.12 ± 26.54	7.40 ± 2.96
*Mycetarotes parallelus*	58.16 ± 30.70 (N = 24)	82.91 ± 46.75	8.60 ± 4.09	66.05 ± 38.05 (N = 18)	74.14 ± 43.75	10.52 ± 3.49
*Mycetophylax morschi* (*restinga*)	57.13 ± 14.48 (N = 15)	69.65 ± 17.98	6.26 ± 1.70	26.53 ± 17.48 (N = 15)	37.53 ± 18.14	3.93 ± 1.14
*Mycetophylax morschi* (dune)	38.50 ± 6.03 (N = 12)	50.15 ± 11.14	5.12 ± 0.63	30.63 ± 8.40 (N = 11)	40.39 ± 12.64	4.92 ± 1.07
*Sericomyrmex parvulus*	87.57 ± 77.97 (N = 19)	93.01 ± 79.60	10.61 ± 8.91	—	—	—
*S. saussurei*	153.92 ± 122.85 (N = 25)	184.86 ± 131.01	15.33 ± 8.81	—	—	—

**Figure 3:**
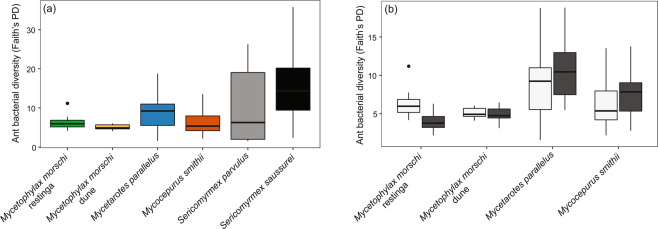
Faith’s Phylogenetic Diversity among: (**a**) the five species of fungus-farming ants from which ants external to the nest were sampled, and (**b**) the three species of fungus-farming ants from which external foragers (white) and internal ants (on the fungus garden; dark grey) were sampled. Note that data for *Mycetophylax morschi* are presented separately for the *restinga* forest and the dune area.

Faith’s Phylogenetic Diversity differed between ant species for which we sampled worker foragers external to the nest (F = 2.81, df = 5, *P* = 0.019; Fig. 3a). *Sericomyrmex saussurei* differed from all other species and *S. parvulus* differed from *Mycetophylax morschi* (dune and *restinga*) and *Mycocepurus smithii* (Fig. 3a). In species for which we sampled individuals internal and external to the nest, Faith’s Phylogenetic Diversity differed between internal and external workers only in *Mycetophylax morschi* from *restinga* (*P* = 0.014; Fig. 3b).

### Composition of bacterial communities: interspecific comparisons and differences between forager and internal ants

**Figure 4:**
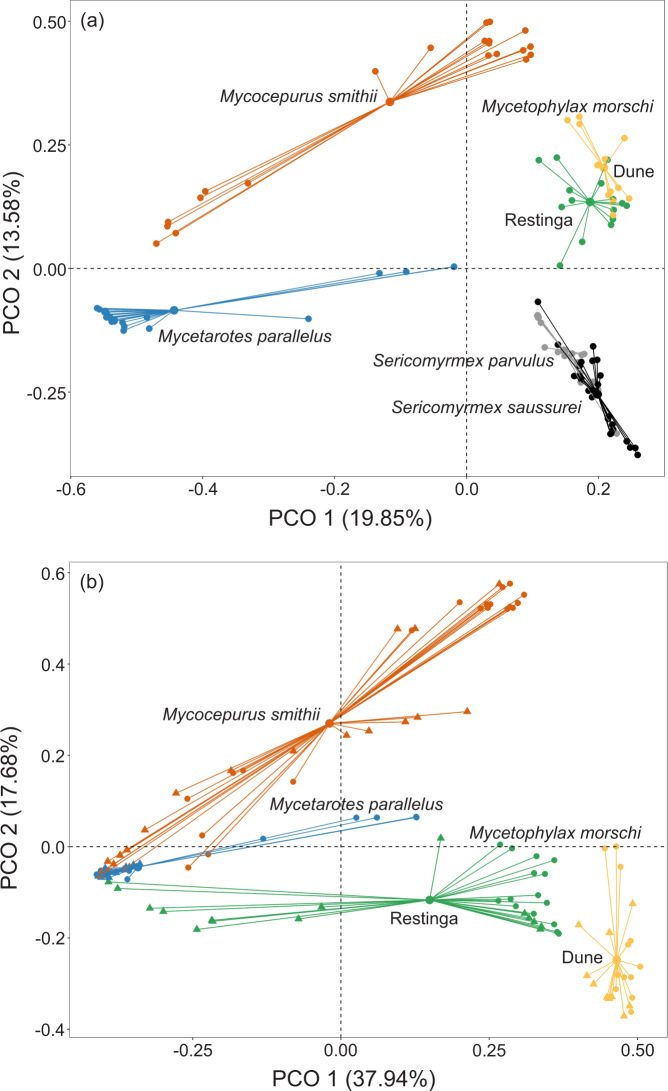
Two-dimensional plot of principal coordinates analysis (Bray-Curtis distance) showing differences in microbiota composition: (**a**) of five species of fungus-farming ants from which ants external to the nest were sampled, and (**b**) of three species of fungus-farming ants from which external and internal ants were sampled. Note that data for *Mycetophylax morschi* are presented separately for the *restinga* forest and the dune area in (**a**) and (**b**). The samples were rarefied at 2000 reads. In (**b**), triangles represent ants internal to the nest (on the fungus garden), and circles represent ants external to the nest (foragers).

The composition of the bacterial communities in individuals external to the nest differed among the attine species (Table 2). Similar patterns were observed when using unweighted and weighted UniFrac distance (Table S2 Supplementary Material 1). We characterized the differences in bacterial composition with the first two axes of a PCoA (Fig. 4a). We also observed that the bacterial composition of external individuals of *Mycetophylax morschi* from *restinga* forest were different from the dune area (Table 2, Fig. 4a).

**Table 2:** Permutational multivariate analysis of variance (PERMANOVA) of bacterial composition using abundance data with Bray-Curtis distances (a) among five species of fungus-farming ants from which we sampled worker foragers external to the nest; and (b) among species from which we sampled ants internal and external to the nest environment.

	df	SS	MS	Pseudo-F	R^2^	*P*(perm)
**(a) All species (ants external to the nest)**						
Species	5	22.202	4.440	19.560	0.470	**0.001**
Residuals	110	24.972	0.227		0.530	
Total	115	47.174				
**Pair-wise tests**	**F model**	**R**^**2**^	***P*** **(adjusted)**			
*Mycetophylax morschi* (*restinga*) *- Mycocepurus smithii*	27.321	0.445	**0.01**			
*Mycetophylax morschi* (*restinga) - Mycetarotes parallelus*	41.973	0.531	**0.01**			
*Mycetophylax morschi* (*restinga*) *- S. saussurei*	15.540	0.290	**0.01**			
*Mycetophylax morschi* (*restinga*) *- S. parvulus*	12.688	0.283	**0.01**			
*Mycocepurus smithii - Mycetarotes parallelus*	26.530	0.381	**0.01**			
*Mycocepurus smithii - S. saussurei*	20.415	0.316	**0.01**			
*Mycocepurus smithii - S. parvulus*	15.352	0.287	**0.01**			
*Mycetarotes parallelus - S. saussurei*	25.204	0.349	**0.01**			
*Mycetarotes parallelus - S. parvulus*	19.092	0.317	**0.01**			
*S. saussurei - S. parvulus*	4.323	0.093	**0.01**			
*Mycetophylax morschi* (*restinga*) *- M. morschi* (dune)	26.296	0.502	**0.01**			
*Mycetophylax morschi* (dune) - *Mycocepurus smithii*	28.031	0.474	**0.01**			
*Mycetophylax morschi* (dune) - *Mycetarotes parallelus*	47.679	0.583	**0.01**			
*Mycetophylax morschi* (dune) - *S. saussurei*	16.594	0.321	**0.01**			
*Mycetophylax morschi* (dune) - *S. parvulus*	13.274	0.314	**0.01**			
**(b) Species with internal and external samples**						
Species	3	19.920	6.640	44.561	0.459	**0.001**
Internal/External	1	1.852	1.852	12.431	0.042	**0.001**
Species * Internal/External	3	2.481	0.827	5.550	0.057	**0.001**
Residuals	128	19.074	0.149		0.440	
Total	135	43.328				
**Pair-wise tests**	**F model**	**R**^**2**^	***P*** **(adjusted)**			
*Mycetophylax morschi* ext - int (*restinga*)	9.616	0.255	**0.02**			
*Mycetophylax morschi* ext – int (dune)	1.063	0.048	1.00			
*Mycocepurus smithii* ext – int	10.441	0.211	**0.02**			
*Mycetarotes parallelus* ext – int	3.644	0.083	0.72			

Statistically significant results are shown in bold. Note that we consider *Mycetophylax morschi* from *restinga* forest separate from the dune area.

When we considered the species of ants for which we sampled individuals internal (on the fungus garden) and external (foragers) to the nest (*Mycocepurus smithii*, *Mycetarotes parallelus*, *Mycetophylax morschi* from the *restinga* forest, and *Mycetophylax morschi* from the dune area), the bacterial community composition differed among the host species, and between foragers and internals within some ant species (Table 2, Fig. 4b. The same pattern was observed with unweighted and weighted UniFrac distance, see Table S2 Supplementary Material 1). In *Mycocepurus smithii* and *Mycetophylax morschi* (*restinga* forest), the bacterial communities of forager and internal workers differed slightly (Table 2, Fig. 4b). Bacterial communities of *Mycetarotes parallelus* and *Mycetophylax morschi* (dune area), however, did not differ between foragers and ants internal to the nest (Table 2, Fig. 4b).

SIMPER results showed that the main OTUs responsible for the dissimilarity between bacterial communities in internal and external workers of *Mycocepurus smithii* were *Chryseobacterium*, *Pseudonocardia* OTU 2, and *Luteimonas*; and in *Mycetophylax morschi* from *restinga* were *Pseudonocardia* OTU 2, *Arthrobacter woluwensis*, Intrasporangiaceae OTU 1, Chitinophagaceae OTU 1, and Nocardioidaceae OTU 1 (See Table S3 Supplementary Material 1). The bacterial communities of internal and external workers of *Mycetarotes parallelus* and *Mycetophylax morschi* from the dune area did not differ significantly.

### Intraspecific differences in bacterial community composition

**Figure 5:**
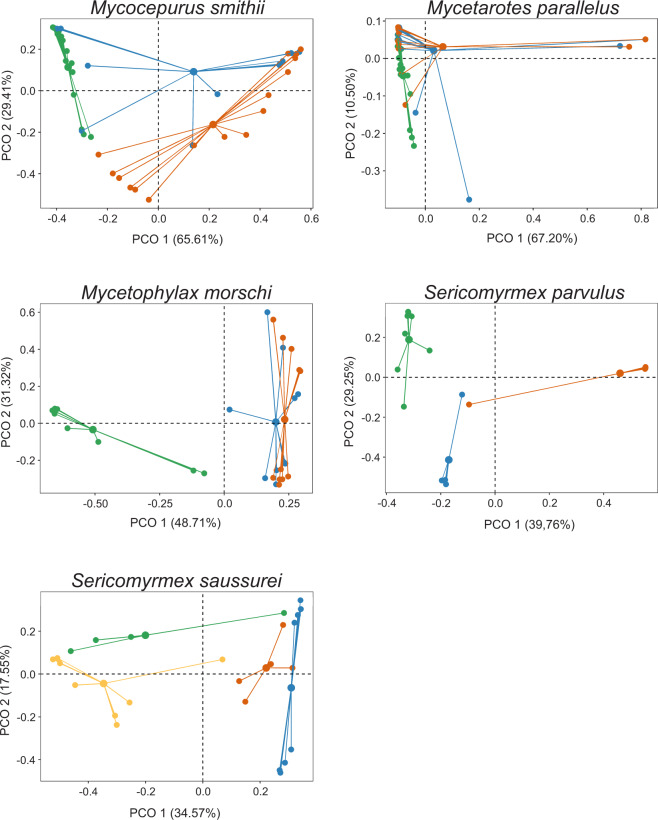
Two-dimensional plot of principal coordinates analysis (Bray-Curtis distance) showing differences in microbiota composition between colonies from each species of fungus-farming ant. The samples were rarefied at 2000 reads. Each color represents one colony. Four nests were sampled for *Sericomyrmex saussurei*. Three nests were sampled for the other species.

The composition of the bacterial community within each species of fungus-farming ant differed among colonies (Table 3, Fig. 5). Specifically, the differences were observed for *Mycocepurus smithii*, *Mycetophylax morschi* from *restinga*, *S. parvulus* and *S. saussurei* (Table 3, Fig. 5). Only *Mycetarotes parallelus* did not show differences in bacterial community composition among colonies (Table 3, Fig. 5).

**Table 3:** Permutational multivariate analysis of variance (PERMANOVA) of bacterial composition (using abundance data with Bray-Curtis distances) between colonies of each species of fungus-farming ant studied in Atlantic rainforest, Brazil.

	df	SS	MS	Pseudo-F	R^2^	*P*(perm)
***Mycocepurus smithii***						
Colony	2	3.442	1.721	12.393	0.394	**0.001**
Residuals	38	5.277	0.138		0.605	
Total	40	8.719				
***Mycetarotes parallelus***						
Colony	2	0.314	0.157	1.623	0.076	0.152
Residuals	39	3.776	0.096		0.923	
Total	41	4.090				
***Mycetophylax morschi***						
Colony	2	3.632	1.816	11.480	0.459	**0.001**
Residuals	27	4.271	0.158		0.540	
Total	29	7.904				
***Sericomyrmex parvulus***						
Colony	2	3.548	1.774	8.241	0.507	**0.001**
Residuals	16	3.444	0.215		0.492	
Total	18	6.992				
***Sericomyrmex saussurei***						
Colony	3	3.226	1.075	4.720	0.402	**0.001**
Residuals	21	4.784	0.227		0.597	
Total	24	8.010				

Statistically significant results are shown in bold.

## Discussion

The current study provided data on the richness and diversity of bacterial communities associated with species of fungus-farming ants (non-leaf-cutters) from the Atlantic rainforest, with novel evidence that microbiota in these ants are host-specific and colony-specific. Additionally, we investigated the potential differences in bacterial communities of ants internal vs. external (foragers) to the nest environment. Our results revealed clear differences in bacterial communities associated with external foragers from the species of fungus-growers, indicating host specificity of these communities. On the other hand, the bacterial communities from internal (on the fungus garden) *versus* external ants did not differ within species (*Mycetarotes parallelus*, *Mycetophylax morschi* from the dune area) or differed only slightly (*Mycocepurus smithii*, *Mycetophylax morschi* from the *restinga* forest). Moreover, the composition of the bacterial community differed among colonies of the same species (with exception of *Mycetarotes parallelus*), which indicates that the bacterial microbiota may be colony-specific.

We showed that the five species of attine ants were predominantly associated with members of the phyla Actinobacteria and Proteobacteria. In a recent study by Sapountzis *et al*.^37^ with 17 species of attine ants (including non-leaf-cutters), the abdominal microbiomes were dominated by Actinobacteria, Proteobacteria and Mollicutes. Actinobacteria and Proteobacteria are frequently present in the fungus garden of leaf-cutters (e.g., *Acromyrmex echinatior*, *Atta cephalotes*, and *Atta colombica*), suggesting a role by these bacteria in nutrient cycling in the fungus-garden^4,11,38^. Actinobacteria comprise a diverse group commonly found in soil-dwelling insects^39^, and are known in attine ants because some of their representatives (e.g., *Pseudonocardia*) produce secondary metabolites with antibiotic activity that protects the fungus garden against pathogens^7,40^. Li *et al*.^16^ recently showed that *Pseudonocardia* bacteria are the dominant filamentous Actinobacteria on the exoskeleton of several species of fungus-farming ants. In our study, the association of *Mycocepurus smithii, Mycetarotes parallelus*, and *Mycetophylax morschi* with Actinobacteria (especially with *Pseudonocardia* in *Mycocepurus smithii* and *Mycetarotes parallelus*) suggests a protective role by these microorganisms that, together with grooming and other hygienic behavior^21^, could reduce pathogenic infection in the fungus garden.

In our study, few OTUs were shared among the five species of fungus-farming ants and each species was dominated by specific OTUs. The microbiota is often host-specific^12,41^, which may be a mechanism to prevent host colonization by pathogenic microorganisms^42^. In fungus-farming ants, the microbiota could be also related with the phylogeny of these ants, as demonstrated by Sapountzis *et al*.^37^. Moreover, bacterial communities in attines could be shaped by ant behaviors, such as grooming and licking, which remove the unwanted bacteria from the cuticle of the ants and from the fungus garden^18,43^. We also suggest that the items collected for fungiculture may influence the bacteria associated with these ants, since different species use variable proportions of vegetable matter and arthropod feces to cultivate the symbiotic fungi^19^. Kellner *et al*.^12^ suggested that some groups of bacteria associated with *Mycocepurus smithii* are influenced by the insect feces collected by the ants. A previous study focusing solely on abdominal microbiomes showed that bacterial communities of *Mycocepurus smithii* vary markedly between field and captive colonies, suggesting that even if host specialization occurs, it is influenced by other factors, such as diet^37^. Overall, we found differences in the phylogenetic diversity of bacteria among species, with *Sericomyrmex saussurei* hosting the highest bacterial diversity. Since *S. saussurei* traveled the greatest foraging distances (up to 3.60 m) among the five studied species^19^, foragers in this species may be exposed to an increased bacterial diversity from the external environment.

Fungus-farming ants species host different bacterial communities, indicating that species identity is an important factor shaping the associated microbiota. Indeed, several studies have shown that host-associated bacterial communities are species-specific^12,37,41,44^. Our findings indicate that bacterial communities differ between host species even when they occur in the same area, suggesting that the ant identity, rather than local microorganism assemblages, drives the composition of associated bacteria. This pattern was also observed for *Megalopta* bees^45^ and Camponotini ants^46,47^. Furthermore, the environment outside the nest seems to have little influence on the associated bacterial community of fungus-farming ants, since the composition of ants on the fungus garden *versus* outside foragers did not differ within species (*Mycetarotes parallelus*, *Mycetophylax morschi* from the dune area) or differed only slightly (*Mycocepurus smithii*, *Mycetophylax morschi* from the *restinga* forest), reinforcing the hypothesis of specificity for this host-bacterial interaction. Similar results were obtained by Kellner *et al*.^12^ for the attine ant *Mycocepurus smithii*, whose associated microbiota was distinct from the community in the soil adjacent to the nest. Ishak *et al*.^48^ also did not observe differences in bacterial community composition between workers of the fungus-farming ant *Trachymyrmex septentrionalis* sampled inside and outside the nest.

Despite the importance of host species identity in predicting associated bacterial communities, environmental characteristics such as temperature, salinity, and pH can also influence the structure of these communities^49,50^. Indeed, we showed that the diversity and composition of bacterial communities associated with *Mycetophylax morschi* differed between *restinga* forest and the dune area. This result may indicate an environmental effect on the bacterial community composition in this species, since *Mycetophylax morschi* from the dune area is constantly under influence of the sea and salinity. Additionally, the dune area has little canopy cover when compared with *restinga* forest (canopy up to 20 m tall)^20^, which could lead to variation in microclimatic conditions near the nests (temperature, humidity). The sampling of a large number of colonies of *Mycetophylax morschi* from the *restinga* forest and dune area coupled with abiotic data (e.g., temperature, humidity, salinity, pH), could clarify if interhabitat difference in associated bacterial communities in this species is affected by the environment or results from intracolonial variation, as detected in the *restinga* forest.

Our results demonstrate that colonies of most species of fungus-farming ants harbor specific bacterial communities, except for *Mycetarotes parallelus*. Ants are social insects that live in populous colonies of related individuals sharing the same space and food^51^, which favors the exchange of microorganisms among nestmates^52^. In other social insects such as bumblebees and termites, the gut microbiota varies among colonies and suggests a colony-specific signature in bacterial communities^53–55^. Hu *et al*.^56^ suggest that differences in bacterial communities between colonies of the same ant species can be explained by host genetic variability, which would lead to a colony-level natural selection of the microorganisms. In addition, the microbiome is related to group-specific odors, suggesting that the members of the same social group harbor similar odor-producing bacterial communities, which is potentially important for within group recognition (e.g., termites^53^, hyenas^57^, meerkats^58^, and the leaf-cutter ant *Acromyrmex echinatior*^59^).

In conclusion, our results show that host species is a strong predictor of the bacterial composition in fungus-farming ants, supporting host species specificity in associated bacterial microbiota. We also detected colony-specificity in the composition of bacterial communities, demonstrating intraspecific variation in the microbiota associated with the attine species investigated. Our study unravels the richness and diversity of bacterial communities that live with fungus-farming ants in the Atlantic rainforest and highlights the importance of describing ant-associated bacterial communities to better understand this host-bacterial interaction in the social environment of insect colonies. The results provide key information for future studies on the ecological and evolutionary processes that drive the success of fungus-farming ants in tropical habitats.

## Supplementary information

Supplementary Material 1.

Supplementary Material 2.

## Data Availability

Ant voucher specimens were deposited at the Museu de Zoologia da Universidade Estadual de Campinas (ZUEC, Campinas, Brazil; registration numbers ZUEC4236 to ZUEC4240). Sequences of all barcoded specimens were deposited in GenBank (accession numbers MH206536- MH206586). Sequences were deposited in the NCBI Sequence Read Archive (accession number PRJNA575362). The dataset supporting this article is available as electronic supplementary material (Supplementary Material 2).
