# Supplementary material for: Symbiotic bacterial communities in rainforest fungus-farming ants: evidence for species and colony specificity
Source: Sci Rep. 2020 Jun 23;10:10172. doi: 10.1038/s41598-020-66772-6 (PMC7311517; doi:10.1038/s41598-020-66772-6)
Supplement: Supplementary file 1 — Supplementary Material 1. [file 41598_2020_66772_MOESM1_ESM.pdf]

Electronic Supplementary Material Scientific Reports

Symbiotic bacterial communities in rainforest fungus-farming ants: evidence for species and colony specificity

Mariane U. V. Ronque[1*], Mariana L. Lyra[2], Gustavo H. Migliorini[3], Maurício Bacci Jr[4] and Paulo S. Oliveira[5]

[1]Programa de Pós-Graduação em Ecologia, Instituto de Biologia, C.P. 6109, Universidade Estadual de Campinas, 13083-862, Campinas - SP, Brazil. ORCID: 0000-0002-2666-2083

[2]Departamento de Zoologia, Instituto de Biociências, Universidade Estadual Paulista - Campus Rio Claro, 13506-900, Rio Claro - SP, Brazil. ORCID: 0000-0002-7863-4965

[3]Programa de Pós-Graduação em Biologia Animal, Universidade Estadual Paulista - Campus São José do Rio Preto, 15054-000, São José do Rio Preto - SP, Brazil. ORCID: 0000-0002-8526-0148

[4]Centro de Estudos de Insetos Sociais, Universidade Estadual Paulista - Campus Rio Claro, 13506-900, Rio Claro - SP, Brazil. ORCID: 0000-0002-5619-1411

[5]Departamento de Biologia Animal, C.P. 6109, Universidade Estadual de Campinas, 13083-862, Campinas - SP, Brazil. ORCID: 0000-0002-4696-2999

[*]Corresponding author: Mariane U. V. Ronque; marianeronque@gmail.com

Table S1. Number of samples and reads for the different subsets of data regarding the five species of fungus-farming ants studied in Brazilian Atlantic rainforest. Here, we do not consider the controls

	No. of total samples (pre/postfiltering)		No. of total reads (pre/postfiltering)	
	Prefiltering	Postfiltering	Prefiltering	Postfiltering
Mycocepurus smithii	47	41	2,496,650	2,377,844
Mycetarotes parallelus	58	42	2,047,571	1,933,576
Mycetophylax morschi	75	53	1,270,783	1,175,446
Sericomyrmex parvulus	32	19	847,872	785,645
Sericomyrmex saussurei	32	25	1,014,913	922,839

Table S2. Permutational multivariate analysis of variance (PERMANOVA) of bacterial composition using the unweighted and weighted UniFrac metrics (a) among five species of fungus-farming ants from which we sampled worker foragers external to the nest; and (b) among species from which we sampled ants internal (on the fungus garden) and external to the nest environment. Statistically significant results are shown in bold. Note that we consider *Mycetophylax morschi* from *restinga* forest separate from the dune area.

	df	SS	MS	Pseudo-F	R^2	*P*(perm)
**(a) All species (ants external to the nest)**						
**Unweighted**						
Species	5	6.432	1.286	5.252	0.192	**0.001**
Residual	110	26.940	0.244		0.807	
Total	115	33.372				
**Weighted**						
Species	5	7.000	1.400	18.359	0.454	**0.001**
Residual	110	8.388	0.076		0.545	
Total	115	15.388				
**(b) Species with internal and external samples**						
**Unweighted**						
Species	3	5.392	1.797	8.007	0.148	**0.001**
Internal/External	1	0.793	0.793	3.533	0.021	**0.001**
Species * Internal/External	3	1.489	0.496	2.210	0.040	**0.001**
Residual	128	28.730	0.224		0.789	
Total	135	36.403				
**Weighted**						
Species	3	5.754	1.918	39.931	0.417	**0.001**
Internal/External	1	0.680	0.680	14.176	0.049	**0.001**
Species * Internal/External	3	1.197	0.399	8.313	0.086	**0.001**
Residual	128	6.148	0.048		0.447	
Total	135	13.781				

Table S3. Similarity percentage analysis (SIMPER) of bacterial communities associated with *Mycocepurus smithii* and *Mycetophylax morschi* (from *restinga* forest), between ants internal (on the fungus garden) and external (foragers) to the nest environment. The table shows the main OTUs that contribute to the dissimilarity of the bacterial communities between internal and external ants. Additionally, we show the abundance of each OTU associated with ants internal and external (foragers) to the nest.

	OTU	Contributions (%)	Cumulative Contributions (%)	**Abundance** External	Internal
Mycocepurus smithii	*Chryseobacterium*	37.1	37.1	21914	2958
	Pseudonocardia OTU2	30.4	67.5	9421	20847
	Luteimonas	22.6	90.1	7840	13326
***Mycetophylax morschi* (*restinga* forest)**	*Pseudonocardia* OTU2	25.0	25.0	1129	12240
	Arthrobacter woluwensis	22.5	47.5	4343	9194
	Intrasporangiaceae OTU1	14.5	62.0	9448	4511
	Chitinophagaceae OTU1	7.1	69.1	3386	28
	Nocardioidaceae OTU1	5.2	74.3	2462	31

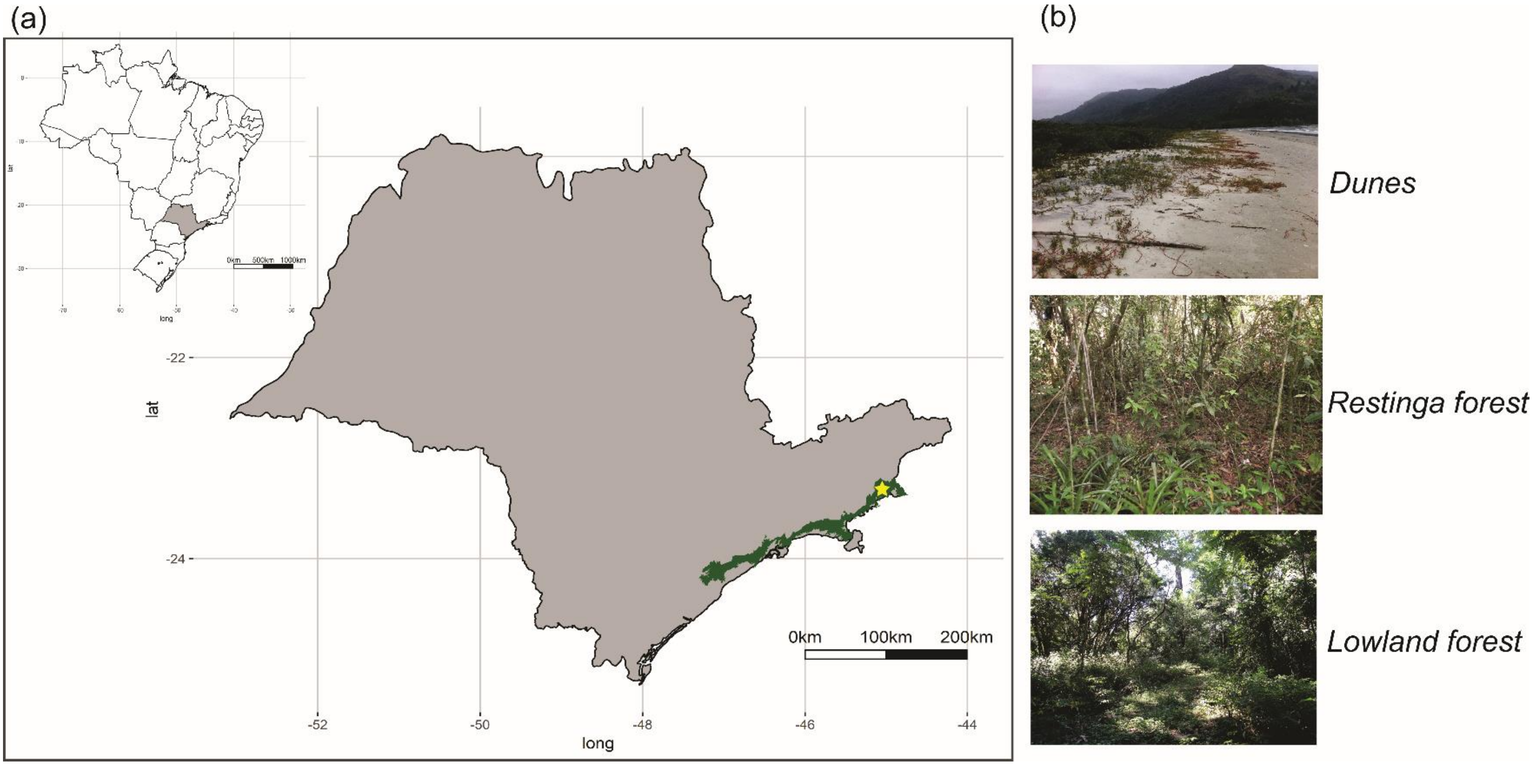

Figure S1. (a) Map showing the State of São Paulo in Brazil (grey) and the 'Parque Estadual Serra do Mar'(green). The study area corresponds to the 'Núcleo Picinguaba' in the North coast of the state and is represented by the yellow star. (b) General view of the three areas where the colonies were sampled: dunes region (*Mycetophylax morschi*), *restinga* forest – coastal sand-based (*Mycocepurus smithii, Mycetarotes parallelus, and Mycetophylax morschi*), and lowland forest (*Sericomyrmex parvulus* and *S. saussurei*). The climate of the region has low thermal amplitude throughout the year and two periods: October to April with frequent rains and maximum precipitation in January, and May to September with fewer rains but without water deficit (San Martin-Gajardo & Morellato 2003). The mean annual rainfall is 2624 mm and the annual average temperature is 21.2 °C, with relative air humidity always exceeding 80%, and with rains throughout the year (San Martin-Gajardo & Morellato 2003). Maps were produced with R version 3.3.3 (R Core Team 2017) with the geographic data available at 'Ministério do Meio Ambiente – Brasil' (http://mapas.mma.gov.br/i3geo/datadownload.htm). Photos: Mariane U. V. Ronque.

Reference: San Martin-Gajardo, I. & Morellato, L. P. Fenologia de Rubiaceae de sub-bosque em floresta Atlântica no sudeste do Brasil. *Revista Brasileira de Botânica* **26**, 299–309 (2003).

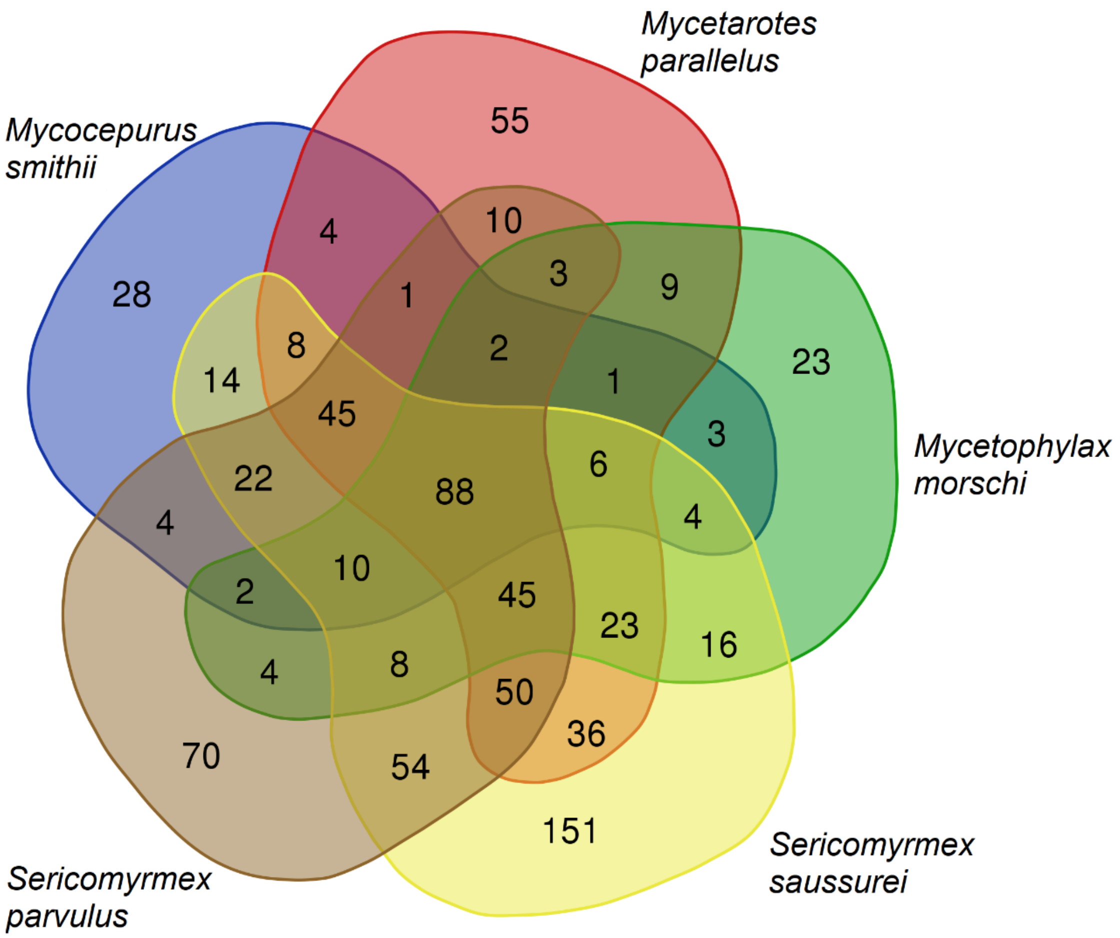

Figure S2. Venn diagrams showing the total number of OTUs shared among the five species of ants from which ants external to the nest (foragers) were sampled.

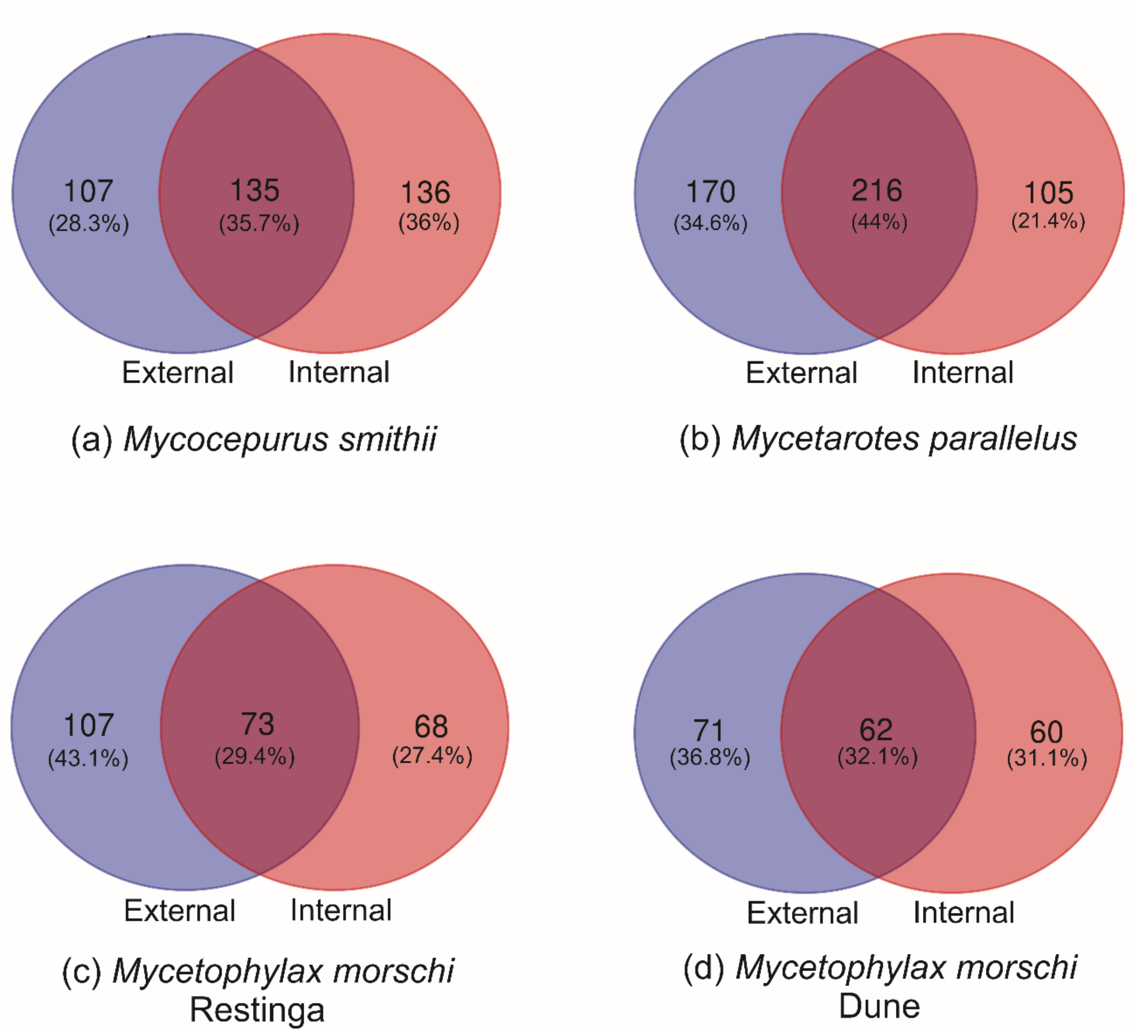

Figure S3. Venn diagrams showing the number of bacterial OTUs that were either shared or exclusive among ants internal (on the fungus garden) or external to the nest (foragers): *Mycocepurus smithii* (a), *Mycetarotes parallelus* (b), *Mycetophylax morschi* from the *restinga* forest (c), and *Mycetophylax morschi* from the dune area (d).
